# Positive Rate of Human Papillomavirus and Its Trend in Head and Neck Cancer in South Korea

**DOI:** 10.3389/fsurg.2021.833048

**Published:** 2022-01-21

**Authors:** Hyun Woong Jun, Yong Bae Ji, Chang Myeon Song, Jae Kyung Myung, Hae Jin Park, Kyung Tae

**Affiliations:** ^1^Department of Otolaryngology-Head and Neck Surgery, College of Medicine, Hanyang University, Seoul, South Korea; ^2^Department of Pathology, College of Medicine, Hanyang University, Seoul, South Korea; ^3^Department of Radiation Oncology, College of Medicine, Hanyang University, Seoul, South Korea

**Keywords:** human papillomavirus, head and neck cancer, squamous cell carcinoma, oropharyngeal cancer, prevalence

## Abstract

**Introduction:**

This study aimed to investigate the positive rate of human papillomavirus (HPV) and its trend in head and neck squamous cell carcinoma (HNSCC) in South Korea and to evaluate the clinical differences between HPV-positive and -negative tumors.

**Methods:**

We studied 300 patients with HNSCC arising in the oropharynx (*n* = 77), oral cavity (*n* = 65), larynx (*n* = 106), hypopharynx (*n* = 40), and sinonasal cavity (*n* = 12), treated in a tertiary university hospital in South Korea from January 2008 to July 2020. HPV status was determined using p16 immunohistochemical staining of formalin-fixed paraffin-embedded tissues.

**Results:**

Of the 300 patients with HNSCC, the positive rate of p16 was 30.3% (91/300). The p16 positive rate was 70.1, 13.9, 20.8, 15, and 0% in the oropharynx, oral cavity, larynx, hypopharynx, and sinonasal cavity, respectively. HPV-positive oropharyngeal squamous cell carcinoma (OPSCC) patients were significantly younger than HPV-negative OPSCC patients. The positive rate of HPV in OPSCC has increased over time from 2008 to 2020, but has not changed significantly in the other primary sites. The disease-free survival curve of HPV-positive OPSCC was significantly better than that of HPV-negative tumors.

**Conclusion:**

The positive rate of HPV in Korean patients with OPSCC is significantly high (70.1%), similar to that in North America and Europe, and has increased abruptly in the past 12 years.

## Introduction

Traditionally, smoking and alcohol drinking have been known to be major causes of head and neck squamous cell carcinoma (HNSCC). Recently, human papillomavirus (HPV) infection emerged as an important cause of HNSCC, especially for oropharyngeal squamous cell carcinoma (OPSCC) (1–5).

HPV is a double-stranded DNA virus that causes abnormal cell proliferation and genomic instability (6). HPV acts as a carcinogen in the cervix, vulva, vagina, penis, anus, and the oropharynx. HPV for head and neck cancer was reported first in 1983 (7). In 2007, the International Agency for Research on Cancer (IARC) recognized HPV as a carcinogen for HNSCC (4). Of more than 200 types of HPV, the most common high-risk type HPV for HNSCC is type 16, followed by type 33 and type 18 (8). As HPV infection is mostly sexually transmitted, an increasing number of HPV-positive HNSCC might be related with changes in sexual behavior (5, 9, 10). HPV-positive OPSCC patients generally tend to be younger and sexually active males, consume less or no alcohol, and are non-smokers (11, 12). Most HPV-positive OPSCC present with early T classification, while often having cystic and multi-level nodal metastases (13). However, HPV-positive OPSCC showed better survival outcomes compared with HPV-negative tumors (5, 14, 15).

The prevalence of HPV in HNSCC is quite diverse, depending on the region and country. In general, the incidence of HPV-related OPSCC has increased over time, and most HPV-unrelated HNSCC decreased or remained stable (2, 16). According to a systematic review study performed in Europe, the positive rate of HPV in OPSCC ranges from 18–65% (17). Another meta-analysis study reported a sharp increase in the proportion of HPV-positive OPSCC up to 72.2% between 2005 and 2009 in North America and Europe (18).

However, the HPV positive rate and recent incidence trend in HNSCC have not been thoroughly investigated in South Korea. Thus, this study aimed to investigate the positive rate of HPV and its trend in HNSCC in South Korea and to evaluate the clinical differences between HPV-positive and HPV-negative HNSCC, especially for OPSCC.

## Materials and Methods

We retrospectively analyzed all patients with HNSCC in the oropharynx, oral cavity, larynx, hypopharynx, and sinonasal cavity treated in a tertiary university hospital in Seoul, South Korea, from January 2008 to July 2020. All patients were diagnosed with squamous cell carcinoma (SCC) pathologically. We excluded patients with nasopharyngeal cancer, salivary cancer, skin cancer, and unknown primary tumors. We also excluded those who had malignant tumors other than SCC, those who had incomplete medical records or were not followed up for at least six months, or those whose formalin-fixed paraffin-embedded tissue blocks were not available (especially patients diagnosed before 2008 because the paraffin blocks were available from 2008 in our institute) from the study. After these exclusions, finally, 300 patients were included and analyzed in this study. This study was approved by the Institutional Review Board (IRB) (IRB No: 2020-07-046).

HPV status of primary tumors was determined using p16 immunohistochemistry (IHC) staining. IHC was performed on 4-μm thick, formalin-fixed, paraffin-embedded tissue sections using the Ventana Benchmark XT automated staining system (Ventana Medical Systems, Tucson, AZ, USA) according to the manufacturer's instructions. Immunohistochemical staining for p16 was performed using the CINtec Histology Kit (clone E6H4, REF 805-4713, Ventana Medical Systems, Tucson, AZ, USA). P16 IHC results were considered positive when there was diffuse moderate or strong nuclear and cytoplasmic staining with grade 2 (moderate) or 3 (strong) intensities and more than 75% distribution in the tumor cells according to the American Joint Commission on Cancer (AJCC) 8th guidelines (19).

We reviewed the medical records and analyzed the demographic and clinicopathological characteristics, including smoking, alcohol consumption, extracapsular spread, TNM stage, treatment methods, recurrence, and survival. TNM staging was categorized according to the American Joint Commission on Cancer (AJCC) Staging Manual 8th edition (19).

SPSS 21.0 (IBM, Armonk, NY) was used for statistical analysis. The chi-square test and Fisher's exact test were used to compare sex, extracapsular spread, smoking, alcohol consumption, TNM staging, and treatment methods between patients with HPV-positive and -negative tumors. A *t-*test was used for age. Statistical significance was set at *P* < 0.05. Overall survival (OS) and disease-free survival (DFS) curves were analyzed over 5 years using the Kaplan-Meier method.

## Results

Of 300 patients with HNSCC, the distribution of the primary site was the oropharynx in 77 patients (25.7%), oral cavity in 65 (21.7%), larynx in 106 (35.3%), hypopharynx in 40 (13.3%), and sinonasal cavity in 12 (4%).

The demographics and clinicopathological characteristics of the patients are summarized in Table 1. Of the 300 patients, 246 were males and 54 were females. The proportion of females was higher in the oral cavity cancer site than in the other primary sites (*P* < 0.001). The mean age was 62.3 ± 11.3 years. The mean age was not significantly different between the five primary sites (*P* = 0.081). Thirty-nine percent of the patients were smokers, and 46% of patients consumed alcohol. The incidence of alcohol consumption was higher in hypopharyngeal cancer than in the four other primary site cancers (*P* = 0.026). The proportion of patients with advanced stage (stage III/IV) was higher in hypopharyngeal and sinonasal cancers (*P* < 0.001). In terms of treatment, the proportion of definitive radiation or concurrent chemoradiation therapy was higher in hypopharyngeal and sinonasal cancers (*P* < 0.001).

**Table 1:** Demographics and characteristics of patients with head and neck squamous cell carcinoma.

	**Oropharynx (*n =* 77)**	**Oral cavity (*n =* 65)**	**Larynx (*n =* 106)**	**Hypopharynx (*n =* 40)**	**Sinonasal cavity (*n =* 12)**	***P* value**
**Age**	60.3 ± 10.0	60.0 ± 14.5	64.8 ± 10.0	64.8 ± 9.6	57.9 ± 10.7	0.081
**Sex**						<0.001
Male	65 (84.4%)	32 (49.2%)	101(95.3%)	37 (92.5%)	11 (91.7%)	
Female	12 (15.6%)	33 (50.8%)	5 (4.7%)	3 (7.5%)	1 (8.3%)	
**HPV**						<0.001
(+)	54 (70.1%)	9 (13.8%)	22 (20.8%)	6 (15.0%)	0 (0.0%)	
(–)	23 (29.9%)	56 (86.2%)	84 (79.2%)	34 (85.0%)	12 (100%)	
**Smoking**						0.813
Non-smoker	47 (61.0%)	43 (66.2%)	60 (56.6%)	24 (60.0%)	7 (58.3%)	
Smoker	30 (39.0%)	22 (33.8%)	46 (43.4%)	16 (40.0%)	5 (41.7%)	
**Alcohol consumption**						0.026
No	40 (51.9%)	42 (64.6%)	53 (50.0%)	16 (40.0%)	10 (83.3%)	
Yes	37 (48.1%)	23 (35.4%)	53 (50.0%)	24 (60.0%)	2 (16.7%)	
**Extracapsular spread**						<0.001
(+)	11 (35.5%)	10 (55.6%)	1 (14.3%)	6 (50.0%)	1 (100%)	
(-)	20 (64.5%)	8 (44.4%)	6 (85.7%)	6 (50.0%)	0 (0.0%)	
**T classification**						<0.001
T1	20 (26.0%)	29 (44.6%)	60 (56.6%)	10 (25.0%)	3 (25.0%)	
T2	38 (49.4%)	20 (30.8%)	15 (14.2%)	10 (25.0%)	2 (16.7%)	
T3	12 (15.6%)	11 (16.9%)	15 (14.2%)	8 (20.0%)	3 (25.0%)	
T4	7 (9.1%)	5 (7.7%)	16 (15.0%)	12 (30.0%)	4 (33.3%)	
**N classification**						<0.001
N0	24 (31.2%)	42 (64.6%)	81 (76.4%)	11 (27.5%)	6 (50.0%)	
N1	29 (37.7%)	9 (13.8%)	9 (8.5%)	11 (27.5%)	4 (33.3%)	
N2	17 (22.1%)	10 (15.4%)	14 (13.2%)	16 (40.0%)	2 (16.7%)	
N3	7 (9.1%)	4 (6.2%)	2 (1.9%)	2 (5.0%)	0 (0%)	
**M classification**						0.170
M0	77 (100%)	65 (100%)	104 (98.1%)	38 (95.0%)	12 (100%)	
M1	0 (0%)	0 (0%)	2 (1.9%)	2 (5.0%)	0 (0%)	
**Stage**						<0.001
I	38 (49.4%)	28 (43.1%)	57 (53.8%)	5 (12.5%)	1 (8.3%)	
II	17 (22.0%)	9 (13.8%)	8 (7.5%)	0 (0%)	4 (33.3%)	
III	10 (13.0%)	10 (15.4%)	14 (13.2%)	6 (15.0%)	2 (16.7%)	
IV	12 (15.6%)	18 (27.7%)	27 (25.5%)	29 (72.5%)	5 (41.7%)	
**Treatment**						<0.001
Surgery	11 (14.3%)	33 (50.8%)	38 (35.8%)	5 (12.5%)	3 (25.0%)	
Surgery + Postop. RT or CCRT	55 (71.4%)	31 (47.7%)	50 (47.2%)	22 (55.0%)	5 (41.7%)	
Definitive RT or CCRT	6 (7.8%)	0 (0.0%)	8 (7.5%)	11 (27.5%)	4 (33.3%)	
Others	5 (6.5%)	1 (1.5%)	10 (9.4%)	2 (5.0%)	0 (0%)	

*HPV, human papilloma virus; RT, radiation therapy; CCRT, concurrent chemoradiation therapy*.

Of the 300 patients, the positive rate of p16 was 30.3% (91/300). The positive rate of p16 was 70.1, 13.9, 20.8, 15, and 0% in the oropharynx, oral cavity, larynx, hypopharynx, and sinonasal cavity, respectively. The HPV-positive rate was significantly higher in OPSCC than in the other primary sites (*P* < 0.05) (Table 1).

Comparisons of HPV-positive and HPV-negative tumors are shown in Table 2. In the oropharynx, the mean age of HPV-positive OPSCC was significantly lower than that of HPV-negative OPSCC (58.6 and 64.2 years in HPV-positive and -negative OPSCC, respectively, *P* = 0.024). The proportion of patients with advanced stage (stage III/IV) was significantly lower in HPV-positive OPSCC than in HPV-negative tumors (*P* < 0.001). Sex, smoking, alcohol consumption, T classification, and N classification were not different between HPV-positive and HPV-negative OPSCC. In the other primary sites, including the oral cavity, larynx, hypopharynx, and sinonasal cavity, all parameters, such as age, sex, smoking, alcohol consumption, TNM stage, and treatment methods did not differ between the HPV-positive and-negative tumors.

**Table 2:** Comparisons of head and neck squamous cell carcinoma according to HPV status.

	**Oropharynx**			**Others**		
	**HPV (+)**	**HPV (–)**	***P*-value**	**HPV (+)**	**HPV (–)**	***P*-value**
**Mean Age**	58.6 ± 9.7	64.2 ± 9.8	0.024	63.6 ± 10.7	62.9 ± 11.9	0.750
**Sex**			0.277			0.365
Male	44 (57.1%)	21 (27.3%)		32 (14.4%)	149 (66.8%)	
Female	10 (13.0%)	2 (2.6%)		5 (2.2%)	37 (16.6%)	
**Years of diagnosis**						
2008–2009	2 (33.3%)	4 (66.7%)		2 (8.3%)	22 (91.7%)	
2010–2011	6 (60.0%)	4 (40.0%)		7 (18.4%)	31 (81.6%)	
2012–2013	10 (76.9%)	3 (23.1%)		10 (27.8%)	26 (72.2%)	
2014–2015	6 (66.7%)	3 (33.3%)		3 (12.0%)	22 (88.0%)	
2016–2017	8 (66.7%)	4 (33.3%)		4 (12.5%)	28 (87.5%)	
2018–2019	12 (80.0%)	3 (20.0%)		8 (14.3%)	48 (85.7%)	
2020	10 (83.3%)	2 (16.7%)		3 (25.0%)	9 (75.0%)	
**Extracapsular spread**			0.472			0.270
(+)	14 (45.2%)	6 (19.4%)		2 (5.3%)	16 (42.1%)	
(–)	9 (29.0%)	2 (6.5%)		5 (13.2%)	15 (39.5%)	
**Smoking**			0.624			0.778
non-smoker	32 (41.5%)	15 (19.5%)		23 (10.3%)	111 (49.8%)	
Smoker	22 (28.6%)	8 (10.4%)		14 (6.3%)	75 (33.6%)	
**Alcohol consumption**			0.974			0.141
No	28 (36.4%)	12 (15.6%)		16 (7.2%)	105 (47.1%)	
Yes	26 (33.8%)	11 (14.3%)		21 (9.4%)	81 (36.3%)	
**T classification**			0.444			0.384
T1/T2	42 (54.5%)	16 (20.8%)		27 (12.1%)	122 (54.7%)	
T3/T4	12 (15.6%)	7 (9.1%)		10 (4.5%)	64 (28.7%)	
**N classification**			0.128			0.647
N0	14 (18.2%)	10 (13%)		22 (9.9%)	118 (52.9%)	
N+	40 (51.9%)	13 (16.9%)		15 (6.7%)	68 (30.5%)	
**Stage**			<0.001			0.834
I&II	49 (63.6%)	6 (7.8%)		18 (8.1%)	94 (42.2%)	
III&IV	5 (6.5%)	17 (22.1%)		19 (8.5%)	92 (41.3%)	
**Treatment**			0.918			0.927
Surgery	7 (9.1%)	4 (5.2%)		13 (5.8%)	66 (29.6%)	
Surgery + Postop RT or CCRT	39 (50.6%)	16 (20.8%)		17 (7.6%)	91 (40.8%)	
Definitive RT or CCRT	4 (5.2%)	2 (2.6%)		4 (1.8%)	19 (8.5%)	
Others	4 (5.2%)	1 (1.3%)		3 (1.3%)	10 (4.5%)	

*HPV, human papilloma virus; RT, radiation therapy; CCRT, concurrent chemoradiation therapy*.

When comparing the four sub-sites of the oropharynx, the positive rate of HPV was 78.4% (40/51) in the tonsil, 61.9% (13/21) in the base of the tongue, 0% (0/1) in the soft palate, and 25% (1/4) in the posterior wall (Table 3).

**Table 3:** Comparisons of oropharyngeal squamous cell carcinoma according to the sub-site.

	**Tonsil (*n* = 51)**			**BOT (*n* = 21)**			**Soft palate (*n* = 1)**			**PPW (*n* = 4)**		
	**HPV (+) (*n =* 40)**	**HPV (–) (*n =* 11)**	***P-value***	**HPV (+) (*n =* 13)**	**HPV (–) (*n =* 8)**	***P-value***	**HPV (+) (*n =* 0)**	**HPV (–) (*n =* 1)**	***P-value***	**HPV (+) (*n =* 1)**	**HPV (–) (*n =* 3)**	***P-value***
**Mean age**	57.9 ± 10.2	64.4 ± 10.6	0.073	60.8 ± 8.3	62.5 ± 11.6	0.694	-	65.0	-	55.0	67.7 ± 2.5	0.049
**Gender**			0.135			0.920			-			-
Male	33 (64.7%)	11 (21.6%)		10 (47.6%)	6 (28.6%)		-	1 (100%)		1 (25.0%)	3 (75.0%)	
Female	7 (13.7%)	0 (0%)		3 (14.3%)	2 (9.5%)		-	-		0 (0.0%)	0 (0.0%)	
**Smoking**			0.290			0.477			-			0.248
Non-smoker	22 (43.1%)	8 (15.7%)		10 (47.6%)	5 (23.8%)		-	-		0 (0.0%)	2 (50.0%)	
Smoker	18 (35.3%)	3 (5.9%)		3 (14.3%)	3 (14.3%)		-	1 (100%)		1 (25.0%)	1 (25.0%)	
**Alcohol consumption**			0.861			0.965			-			0.046
No	23 (45.1%)	6 (11.8%)		5 (23.8%)	3 (14.3%)		-	-		0 (0.0%)	3 (75.0%)	
Yes	17 (33.3%)	5 (10.0%)		8 (38.1%)	5 (23.8%)		-	1 (100%)		1 (25.0%)	0 (0.0%)	
**T classification**			0.470			0.776			-			0.505
T1/T2	33 (64.7%)	8 (15.7%)		9 (42.9%)	6 (28.6%)		-	1(100%)		0 (0%)	1 (25%)	
T3/T4	7 (13.7%)	3 (5.9%)		4 (19%)	2 (9.5%)		-	-		1 (25%)	2 (50%)	
**N classification**			0.256			0.920			*-*			0.248
N0	11 (21.6%)	5 (9.8%)		3 (14.3%)	2 (9.5%)		-	1 (100%)		0 (0%)	2 (50%)	
N+	29 (56.9%)	6 (11.8%)		10 (47.6%)	6 (28.6%)		-	-		1 (25%)	1 (25%)	
**Stage**			<0.001			0.006						-
I&II	38 (74.5%)	3 (5.9%)		11 (52.4%)	2 (9.5%)		-	1 (100%)		-	-	
III&IV	2 (3.9%)	8 (15.7%)		2 (9.5%)	6 (28.6%)		-	-		1 (25%)	3 (75%)	
**Treatment**			0.596			0.072			*-*			0.135
Surgery	5 (9.8%)	1 (2%)		2 (9.5%)	3 (14.3%)		-	-		-	-	
Surgery + Postop RT or CCRT	32 (62.7%)	10 (19.6%)		7 (33.3%)	3 (14.3%)		-	1 (100%)		-	2 (50%)	
Definitive RT or CCRT	3 (5.9%)	0 (0%)		0 (0%)	2 (9.5%)		-	-		1 (25%)	-	
Others	-	-		4 (19.0%)	-		-	-		-	1 (25%)	

*BOT, base of tongue; PPW, posterior pharyngeal wall; HPV, human papilloma virus; RT, radiation therapy; CCRT, concurrent chemoradiation therapy*.

**Figure 1:**
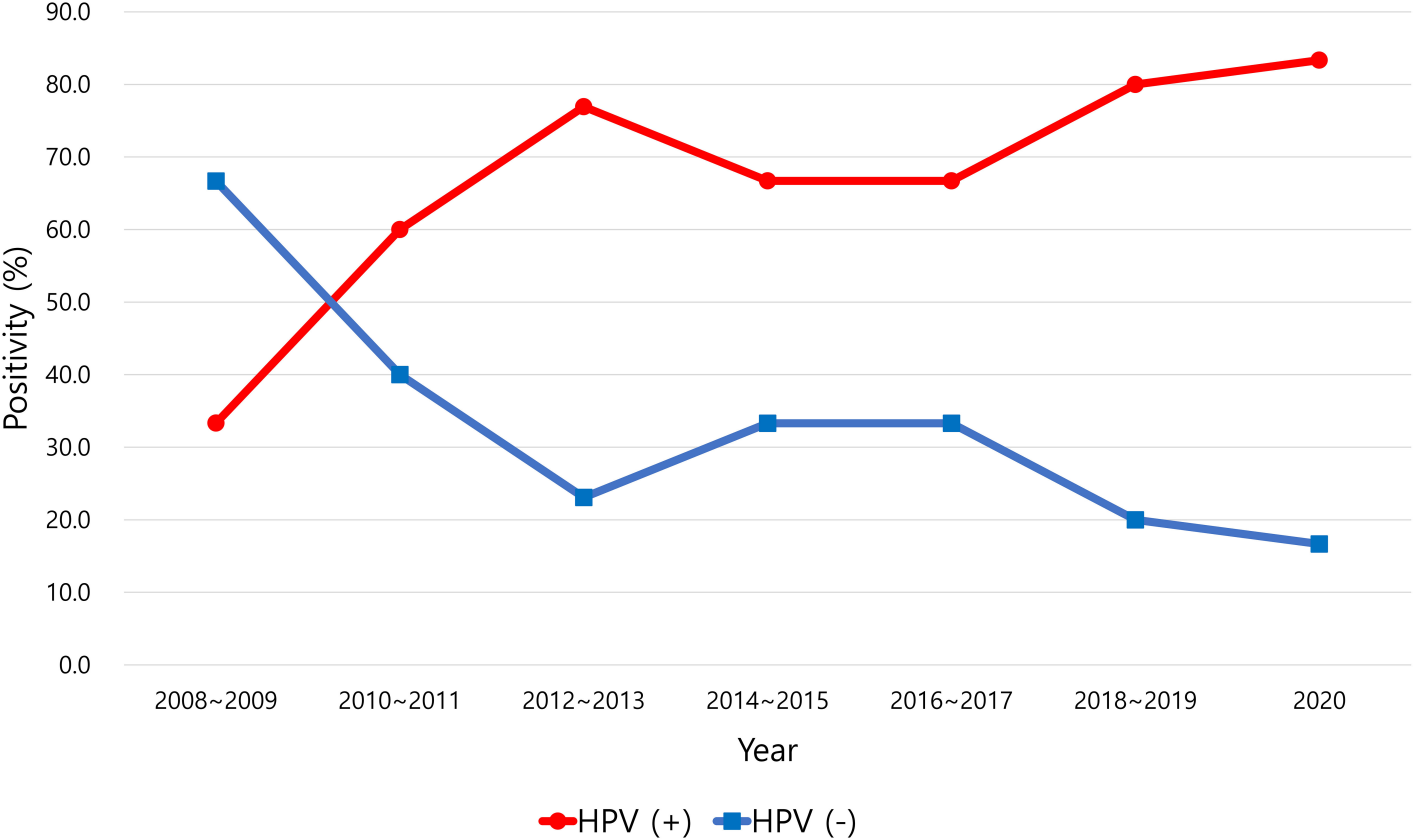
Positive rate of human papillomavirus (HPV) in oropharyngeal squamous cell carcinoma.

**Figure 2:**
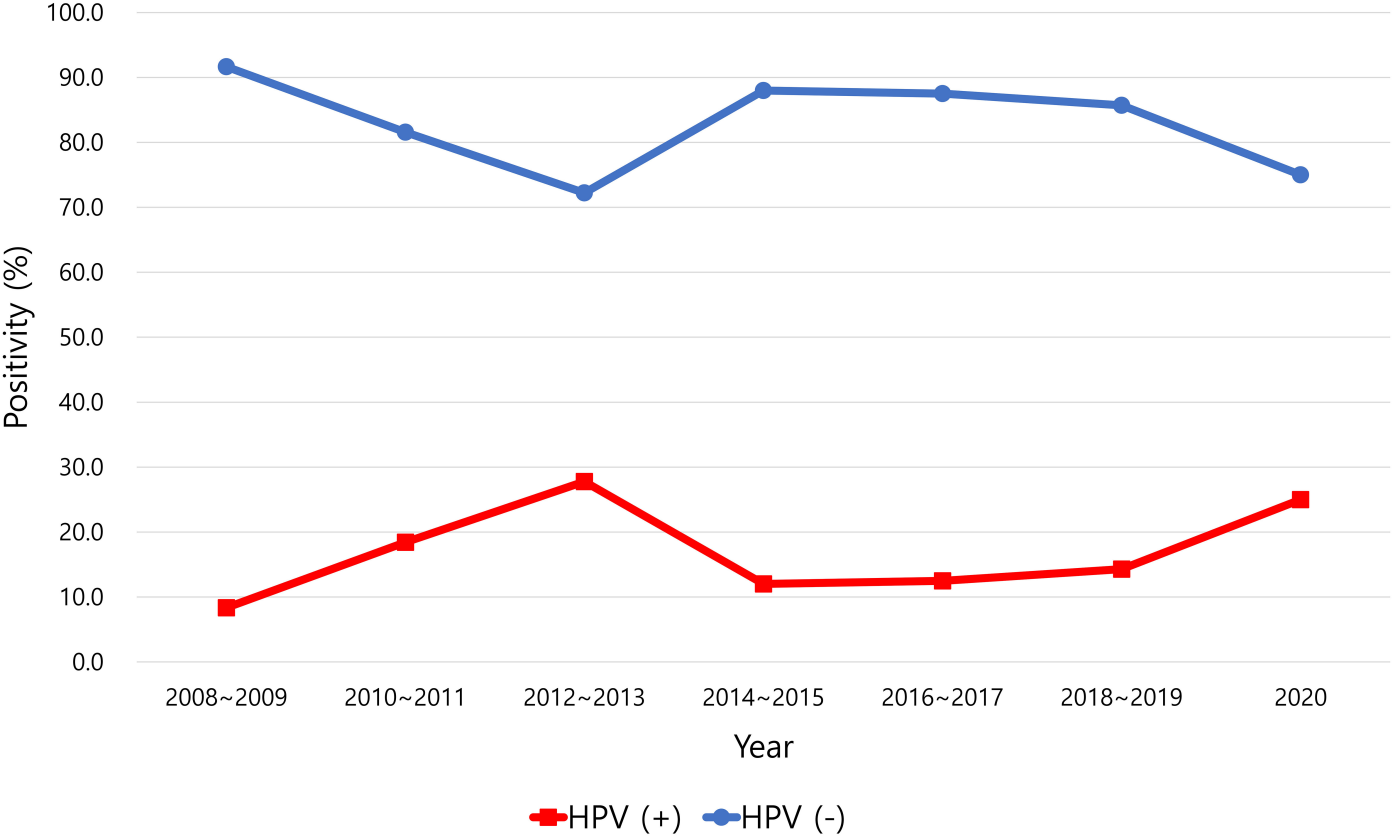
Positive rate of human papillomavirus (HPV) in squamous cell carcinoma of the oral cavity, larynx, hypopharynx, and sinonasal cavity.

HPV positivity in OPSCC tended to increase over time from 2008 to 2020 (Figure 1). The positive rate of HPV infection in OPSCC was 33.3% in 2008–2009 and increased to 83.3% in 2020. The incidence of HPV-positive and HPV-negative OPSCC was reversed between 2009 and 2010 (Figure 1). In the other primary sites, the positive rate of HPV did not change significantly over the years (Figure 2).

**Figure 3:**
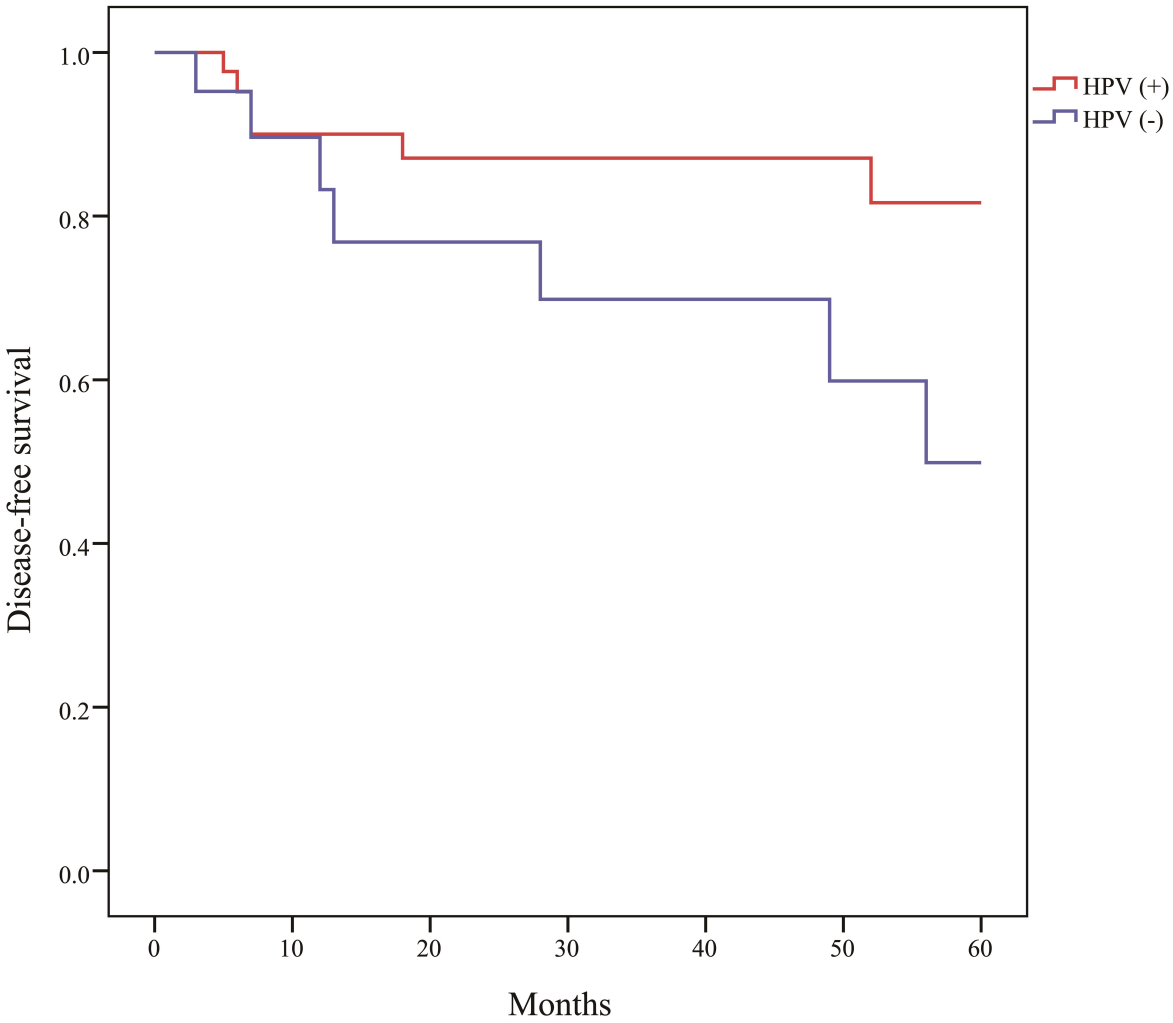
Disease-free survival of oropharyngeal squamous cell carcinoma according to human papillomavirus (HPV) status (*P* = 0.028).

The median follow-up time after treatment was 34 months (range, 6–124 months). The Kaplan-Meier curve of DFS of HPV-positive OPSCC was significantly higher than that of HPV-negative OPSCC (Figure 3). The OS curves were not statistically different between HPV-positive and HPV-negative OPSCC, although the OS of HPV-positive OPSCC tended to be higher than that of negative tumors. The DFS and OS curves did not differ between HPV-positive and HPV-negative tumors in patients with oral cancer, laryngeal cancer, and hypopharyngeal cancer. According to the stage, the DFS of stage I/II disease was significantly higher than that of stage III/IV disease in OPSCC and cancer of other primary sites.

## Discussion

The prevalence of HPV in OPSCC is rising worldwide, starting from developed Western countries, and it differs significantly across different countries and cultures (16, 20). The well-known high-burden regions for HPV in OPSCC are North America and northern Europe, including the United States [81.6% (21), 74% (22), 67% (23), 66% (24), 65% (25), and 58% (26)], Canada (57%) (27), Sweden [68% (28), 70% (29), and 85% (30)], Denmark (52%) (31), France [46.5% (32) and 50.4% (33)], and Norway [52% (34) and 77% (35)].

Southeast Europe is known to be a relatively low-burden region in Western countries. However, a study conducted in Greece showed an increased rate of HPV-positive OPSCC in 2000–2007 (50%) compared to that in 1992–1998 (17%) (36). A recent increased incidence of HPV-positive OPSCC was also reported in Spain from 8.2% in 2007–2011 to 21.5% in 2012–2016 (37). Another study conducted in Italy also showed an increase in HPV-positive OPSCC from 16.7% in early 2000–2006 to 46.2% in 2013–2018 (38). In Australia, the positivity rate of HPV in OPSCC increased from 20.2% in 1987–1995 to 63.5% in 2006–2010 (10). In Brazil, a study using a birth cohort revealed an increase in HPV-related oropharyngeal/oral cancer in young males and females (12).

Asia has a relatively lower prevalence of HPV-positive OPSCC, although the incidence has been increasing in recent years. One study revealed an increasing incidence of HPV in Thailand, with an annual increase of 2% from 16% in 2012 to 26% in 2017 (39). The HPV-positive rate of OPSCC in Asia was reported to be 28% in Thailand (40), 12.6% in Taiwan (41), 22.8% in India (42), 39% in southern China (43), 45% in Singapore (44), and 44% in Japan (45).

In this study, the HPV-positive rate of OPSCC was 70.1%, and the positive rate showed a dramatic increase from 33.3% in 2008–2009 to 80% in 2020. HPV-positive OPSCC outnumbered HPV-negative OPSCC in 2009–2010. The recent positive rate of HPV in OPSCC in South Korea seems to be similar to that in Western countries. Chaturvedi et al. revealed a rising incidence of HPV-positive OPSCC in the United States, from 16.3% in the 1980s to 71.7% in the 2000s (9). In their study, HPV-positive OPSCC outnumbered HPV-negative OPSCC in the mid-1990s (9). The time gap when HPV-positive OPSCC overtakes HPV-negative OPSCC is approximately 15 years between the United States and South Korea.

In previous studies performed in South Korea for patients in 1994–2010, the positive rate of HPV in OPSCC ranged from 23.4 to 49.5% (46–50). In a recent study conducted in South Korea, the positive rate of HPV was 73% by p16 IHC in patients with OPSCC between 2011 and 2019, similar to that shown in this study (51). Recently, Jung et al. performed a study using the Korea Central Cancer Registry to evaluate the recent incidence trend of head and neck cancer (52). They reported increased HPV-related oropharyngeal cancer and a slight decrease in HPV-unrelated head and neck cancer. Interestingly, the incidence of tonsillar cancer increased continuously from 1999 to 2011 but seemed to stabilize thereafter until 2020 (52). However, they did not evaluate the HPV status in their study. Therefore, they could not confirm that the increase in tonsillar cancer was associated with increased HPV-positive cancer. The current study showed that the positive rate of HPV in OPSCC tended to increase over time from 2008 to 2020, similar to the increase in oropharyngeal cancer in Jung's study. Therefore, this study might suggest that the increased incidence of OPSCC in South Korea is associated with an increase in HPV-positive tumors.

Generally, the positive rate of HPV in the other primary sites is low compared to that in the oropharynx. In this study, HPV positivity was 13.9, 20.8, and 15% in the oral cavity, larynx, and hypopharynx, respectively. The incidence of HPV positivity in oral cancer was reported to be 26% in Croatia (53), 31% in India (42), 6.9% in the United States (54), 10.5% in France (32), 3.4% in Brazil (55), and 0.6% in South Korea (51). The HPV-positive rate of laryngeal cancer was also diverse across countries. It was 4% in Mexico (56), 7.2% in Thailand (39), 9% in Italy (57), 10.6% in Austria (58), 11.6% in South Korea (51), 20% in Turkey (59), 25.8% in China (43), and 60% in Spain (60). The HPV positivity of hypopharyngeal cancer varied from 5% (61) and 21.6% (62) in the United States, 4.1% in Austria (58), 5% in Spain (60), 16.5% in Sweden (63), and 10% in South Korea (64). An international multicenter study performed in 2016, including 29 countries, reported a 3.9% HPV positivity rate in hypopharyngeal cancer (65).

In sinonasal cancer, the rate of HPV positivity was 20% in India (66), 23% (67) and 53% (68) in the United States, 15.7% in Eastern China (69), and 28% in South Korea (51). The current study revealed 0% (0/12) of HPV positivity in sinonasal cancer, which is significantly different from that found in other studies. This might be associated with the small sample size of the 12 patients in this study. Further studies with a larger sample size are necessary to clarify the positive rate of HPV in sinonasal cancer.

Currently, there is no standard method to examine the HPV status of clinical cancer samples. The targets for HPV testing might include p16 protein, HPV DNA or mRNA, viral oncoproteins, and HPV-specific serum antibodies (6). The reliable and effective tool is determined by accuracy, feasibility, and cost-effectiveness. The method of directly detecting viral DNA using polymerase chain reaction (PCR) has high sensitivity and can identify high-risk HPV (70). *In situ* hybridization (ISH) enables direct identification of HPV in topographical relationship to tumor cells (70). However, the most popular and cost-effective method for the detection of HPV is p16 IHC. The overexpression of p16 is a surrogate indicator of HPV infection (70). The eighth edition of the AJCC cancer staging guidelines recommends p16 IHC as a standard marker for HPV infection in OPSCC (19). However, p16 IHC might not be sufficient as a single marker for detection of HPV infection because approximately 10–15% of p16 positive tumors do not reveal the presence of HPV in ISH or PCR testing for the virus (71, 72). This study evaluated HPV status using only p16 IHC, which is more widely used clinically than HPV PCR or ISH. Therefore, caution should be exercised when interpreting the results of this study.

It is known that the survival rate of HPV-positive OPSCC is better than that of HPV-negative tumors (5, 14, 15, 35). This study confirmed that the DFS of HPV-positive OPSCC is significantly better than that of HPV-negative tumors (*P* = 0.028), similar to that reported in previous studies.

This study had some limitations. First, it was a retrospective study conducted in a single institute, and this study included a relatively small sample size. Therefore, selection bias is inevitable. The results of this study may not represent the actual HPV status of HNSCC in South Korea. Second, we performed only p16 IHC staining without HPV genotyping to analyze the HPV status. Therefore, HPV status might not be determined precisely, although the AJCC guidelines recommend p16 IHC as the standard method. A further multicenter study with a larger study sample and additional HPV genotyping is necessary to overcome the limitations of this study.

Despite these limitations, it is noteworthy that this study is the first to show a significant increase in HPV positivity in OPSCC in South Korea during the past 12 years, close to the positive rate found in Western countries.

## Conclusions

The positive rate of HPV was significantly higher in OPSCC than in other head and neck primary site cancers in South Korea. The HPV positivity of OPSCC has been increasing abruptly during the past 12 years, and it is becoming similar to the positive rate found in North America and Europe. The DFS of HPV-positive OPSCC was significantly better than that of HPV-negative tumors.

## Data Availability Statement

The original contributions presented in the study are included in the article/supplementary material, further inquiries can be directed to the corresponding author/s.

## Ethics Statement

The studies involving human participants were reviewed and approved by the Institutional Review Board (IRB) of Hanyang University Hospital. The patients/participants provided their written informed consent to participate in this study.

## Author Contributions

HJ: initial draft, data acquisition, analysis, critical revision of manuscript, and final approval. YJ, CS, JM, HP: data acquisition, analysis, critical revision of manuscript, and final approval. KT: conception and design of the work, data analysis and interpretation, critical revision of manuscript, and final approval. All authors contributed to the article and approved the submitted version.

## Conflict of Interest

The authors declare that the research was conducted in the absence of any commercial or financial relationships that could be construed as a potential conflict of interest.

## Publisher's Note

All claims expressed in this article are solely those of the authors and do not necessarily represent those of their affiliated organizations, or those of the publisher, the editors and the reviewers. Any product that may be evaluated in this article, or claim that may be made by its manufacturer, is not guaranteed or endorsed by the publisher.
